# An evaluation of the PacBio RS platform for sequencing and *de novo* assembly of a chloroplast genome

**DOI:** 10.1186/1471-2164-14-670

**Published:** 2013-10-01

**Authors:** Marco Ferrarini, Marco Moretto, Judson A Ward, Nada Šurbanovski, Vladimir Stevanović, Lara Giongo, Roberto Viola, Duccio Cavalieri, Riccardo Velasco, Alessandro Cestaro, Daniel J Sargent

**Affiliations:** 1 Research and Innovation Centre, Fondazione Edmund Mach, Via E. Mach 1, 38010 San Michele all’Adige, Italy; 2 Driscoll’s, 151 Silliman Road, Watsonville, CA 95077-5045 USA; 3 Institute of Botany and Botanical Garden, Faculty of Biology, University of Belgrade, Takovska 43, 11000 Belgrade, Serbia

**Keywords:** Third-generation sequencing, NGen, Genomics, Assembly, Annotation, Oxford nanopore, Pacific BioSciences, Roche 454

## Abstract

**Background:**

Second generation sequencing has permitted detailed sequence characterisation at the whole genome level of a growing number of non-model organisms, but the data produced have short read-lengths and biased genome coverage leading to fragmented genome assemblies. The PacBio RS long-read sequencing platform offers the promise of increased read length and unbiased genome coverage and thus the potential to produce genome sequence data of a finished quality containing fewer gaps and longer contigs. However, these advantages come at a much greater cost per nucleotide and with a perceived increase in error-rate. In this investigation, we evaluated the performance of the PacBio RS sequencing platform through the sequencing and *de novo* assembly of the *Potentilla micrantha* chloroplast genome.

**Results:**

Following error-correction, a total of 28,638 PacBio RS reads were recovered with a mean read length of 1,902 bp totalling 54,492,250 nucleotides and representing an average depth of coverage of 320× the chloroplast genome. The dataset covered the entire 154,959 bp of the chloroplast genome in a single contig (100% coverage) compared to seven contigs (90.59% coverage) recovered from an Illumina data, and revealed no bias in coverage of GC rich regions. Post-assembly the data were largely concordant with the Illumina data generated and allowed 187 ambiguities in the Illumina data to be resolved. The additional read length also permitted small differences in the two inverted repeat regions to be assigned unambiguously.

**Conclusions:**

This is the first report to our knowledge of a chloroplast genome assembled *de novo* using PacBio sequence data. The PacBio RS data generated here were assembled into a single large contig spanning the *P. micrantha* chloroplast genome, with a higher degree of accuracy than an Illumina dataset generated at a much greater depth of coverage, due to longer read lengths and lower GC bias in the data. The results we present suggest PacBio data will be of immense utility for the development of genome sequence assemblies containing fewer unresolved gaps and ambiguities and a significantly smaller number of contigs than could be produced using short-read sequence data alone.

## Background

The ability to perform sequencing and *de novo* assembly of genomes has been greatly facilitated in recent years thanks to the advent of second-generation sequencing technologies, and as such is becoming relatively routine for genome analysis of all but the largest and most complex genomes. The range of platforms available for sequencing is increasing, and novel ‘third-generation’ technologies promising advantages over the more established ‘second-generation’ short read sequencing platforms have recently been brought to market.

The ‘second-generation’ sequencing revolution, which began with the release of the 454 pyro-sequencing platform [1], has been dominated in recent years by Illumina, who deliver up to 600 Gb of sequence data per run with the HiSeq2500. Illumina’s technology employs sequencing-by-synthesis [2] in which fluorescently labelled reversible terminator nucleotides are imaged as they are incorporated into growing DNA strands synthesised from clonally amplified DNA templates that are immobilised onto the surface of a glass flow-cell. The HiSeq platform has become the industry standard for high throughput DNA sequencing in terms of throughput and accuracy; however, the technology is limited by the number of nucleotides that can be sequenced from a given DNA template, currently less than ~250 bases, and amplification of the DNA template by PCR is typically required before sequencing, leading to a base-composition bias in genome coverage due to the chemical-physical properties of the DNA template [3].

Recently, Pacific Biosciences released their PacBio RS sequencing platform which offers real-time sequencing from single polymerase molecules [4]. The procedure, termed single-molecule real-time (SMRT) sequencing, utilises DNA polymerase molecules bound to 50 nm-wide nanophotonic structures in an array slide which Pacific Biosciences have called ‘zero-mode waveguides’ (ZMWs). The polymerases synthesise DNA from a template using four fluorescently-labelled nucleotides within the ZMWs and thus sequencing requires no prior amplification of the DNA template. The width of the ZMWs permits light to enter and excite the fluorophore that is being incorporated into the growing DNA strand, but not to propagate through the wave-guide, enabling single-fluorophore detection simultaneously in each ZMW on the array in real-time as the DNA strand is synthesised. The data produced from the ‘third-generation’ PacBio RS sequencing platform has a significantly longer read length than that of ‘second-generation’ technologies such as the Illumina HiSeq2000, and maximum read lengths of 23,000 bp have been reported in the literature, with current average read lengths reaching 2,246 kbp [5]. However, the raw data generated from the PacBio RS platform is inherently error-prone, with up to 17.9% errors having been reported [6], the majority being indel events, caused by incorporation events or the intervals between them being too short to be reliably detected [4]. Despite this drawback, context-specific error modes affecting short-read sequencing platforms [7] are nearly absent from PacBio data. Instead, the error model of PacBio data is random, and thus with sufficient depth of coverage, up to 99.9% consensus accuracy can be achieved from sequencing and *de novo* assembly using PacBio RS sequencing data [8]. This lack of context-specific error combined with PacBio’s long single-molecule derived reads has allowed sequencing through both plant and animal long tandem repeats [9], which are very difficult to resolve with any other platform. Additionally, the recent release of the hierarchical genome assembly process (HGAP) workflow of the SMRT-analysis pipeline [10] permits error-correction of continuous long reads to be performed without the need for additional circular consensus PacBio sequencing data, or short-read sequencing data from other platforms.

Mitochondrial and chloroplast genomes make interesting targets for evaluation of the PacBio system because despite the fact that plastid genomes are relatively small, they are rarely completely assembled from second generation sequencing technologies unless specifically targeted, and even then assemblies are often fragmented into relatively large numbers of contigs even at high levels of coverage [11]. Assembly of plastid genomes with PacBio data would also allow for the evaluation of the platform to resolve long inverted repeats that are characteristic of chloroplast genomes and which are difficult to resolve with other sequencing platforms.

In this investigation, the performance of the PacBio RS sequencing platform for the sequencing and *de novo* assembly of the chloroplast genome of a member of the Rosaceae, *Potentilla micrantha*, was evaluated. To our knowledge this is the first report of a chloroplast genome sequenced using PacBio RS data. Since data generated using the Illumina HiSeq2000 platform are considered to be of very high quality, the relative performance of the PacBio sequence data was evaluated in relation to a *de novo* assembly of the same genome performed with data generated from a single Illumina library sequenced on a single lane of Illumina HiSeq2000. The performance of the data generated from the PacBio RS platform is discussed.

## Results

### Data output from Illumina HiSeq2000 and PacBio RS platforms

Following extraction of reads containing only chloroplast genome sequence data and prior to error-correction, PacBio RS reads with a mean length of 3,936.66 bp were recovered, totalling 223,483,907 nucleotides. Post HGAP error-correction [10] (see Methods section), 28,638 PacBio RS reads were recovered with a mean read length of 1,902.75 bp totalling 54,492,250 bp. Following trimming, 7,164,496 paired Illumina reads with a mean length of 99.22 bp were recovered containing a total of 1,421,726,349 nucleotides.

### Assembly of the chloroplast genome sequence

#### PacBio RS

A total of 97 overlapping contigs were obtained from the Celera assembly of the chloroplast reads of the HGAP-corrected PacBio dataset, which were merged into a single contiguous sequence using minimus2 and SeqMan (Lazergene). The PacBio contig contained a total of 139,688 nucleotides. The two IRs in the PacBio dataset differed at three nucleotide positions which allowed the two IRs to be resolved across 10,259 nucleotides. The remaining 15,271 bp section of the inverted repeat (IR) was identical in both IRs and thus the total length for the *P. micrantha* chloroplast genome was 154,959 bp.

#### Illumina HiSeq2000

**Figure 1:**
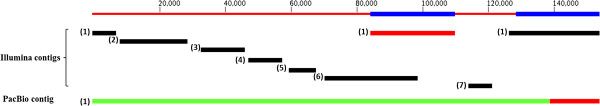
**Sequence data coverage of the *P. micrantha *chloroplast genome.** Schematic diagram showing the coverage of the *P. micrantha* chloroplast genome by the seven Illumina contigs (black) and a single PacBio contig (green) following assembly using ABySS and Celera assembler respectively. The red line across the top of the schematic represents the *P. micrantha* chloroplast genome sequence, blue bold sections indicate the inverted repeat regions of the genome. Sections of contig 1 from both the Illumina and PacBio assemblies corresponding to the non-unique section of the IR are shown in red. Illumina contig 1 spans the start/end point of the linear representation of the circular chloroplast genome.

The chloroplast reads extracted from the Illumina dataset were assembled into a total of seven contigs containing 114,841 nucleotides, including a single 25,530 bp inverted repeat (IR). Since the chloroplasts of angiosperms contain a large sequence repeated once in reverse polarity [12], the sequence was resolved manually based on read depth within the region and comparison to the IR of the *Fragaria* chloroplast genome to identify IR borders (see Methods), in line with the methodology used to defined the chloroplast genomes of other plant species [13], to give a total length of 140,371 bp (Figure 1). Contigs had a minimum length of 6,908 bp, a maximum length of 35,424 and a mean and N50 length of 17,606 and 30,422 respectively. The gaps in the Illumina assembly had a minimum length of 239 bp, a maximum length of 5431 and a mean length of 2084 bp. The average GC content of the gaps in the assembly was 14.63%, compared to an average GC content of the chloroplast consensus sequence of 37.22%.

A summary of the data generated and the assemblies produced from the PacBio RS data in comparison to the data generated from the Illumina HiSeq2000 platform is given Table 1.

**Table 1:** ***P. micrantha *chloroplast sequencing data statistics**

	**PacBio RS**	**Illumina HiSeq2000**
Number of raw reads reads^1^	56,770	7,164,496 (paired reads)
Total nucelotides (raw data)^1^	223,483,907	1,421,726,349
Mean read length (raw data)^1^	3,937	99
Total nucleotides (error-corrected data)	54,492,250	n.a.
Mean read length (error-corrected data)	1,902	n.a.
Pre-assembly error-rate^2^	1.3%	0.117%
Ambiguous bases post-assembly^3^	0%	0.12%
Assembled genome coverage	100%	90.59%
Average depth of coverage	320×	9,111×
Number of contigs	1	7
Total genome coverage (bp)	154,959	148,776

Summary statistics for the assembly of the *P. Micrantha* chloroplast genome using PacBio RS and Illumina HiSeq2000 sequencing data.

^1^Trimmed Illumina reads.

^2^Error-corrected PacBio reads and raw Illumina reads.

^3^In comparison to the chloroplast consensus sequence.

### Depth of coverage and GC bias

**Figure 2:**
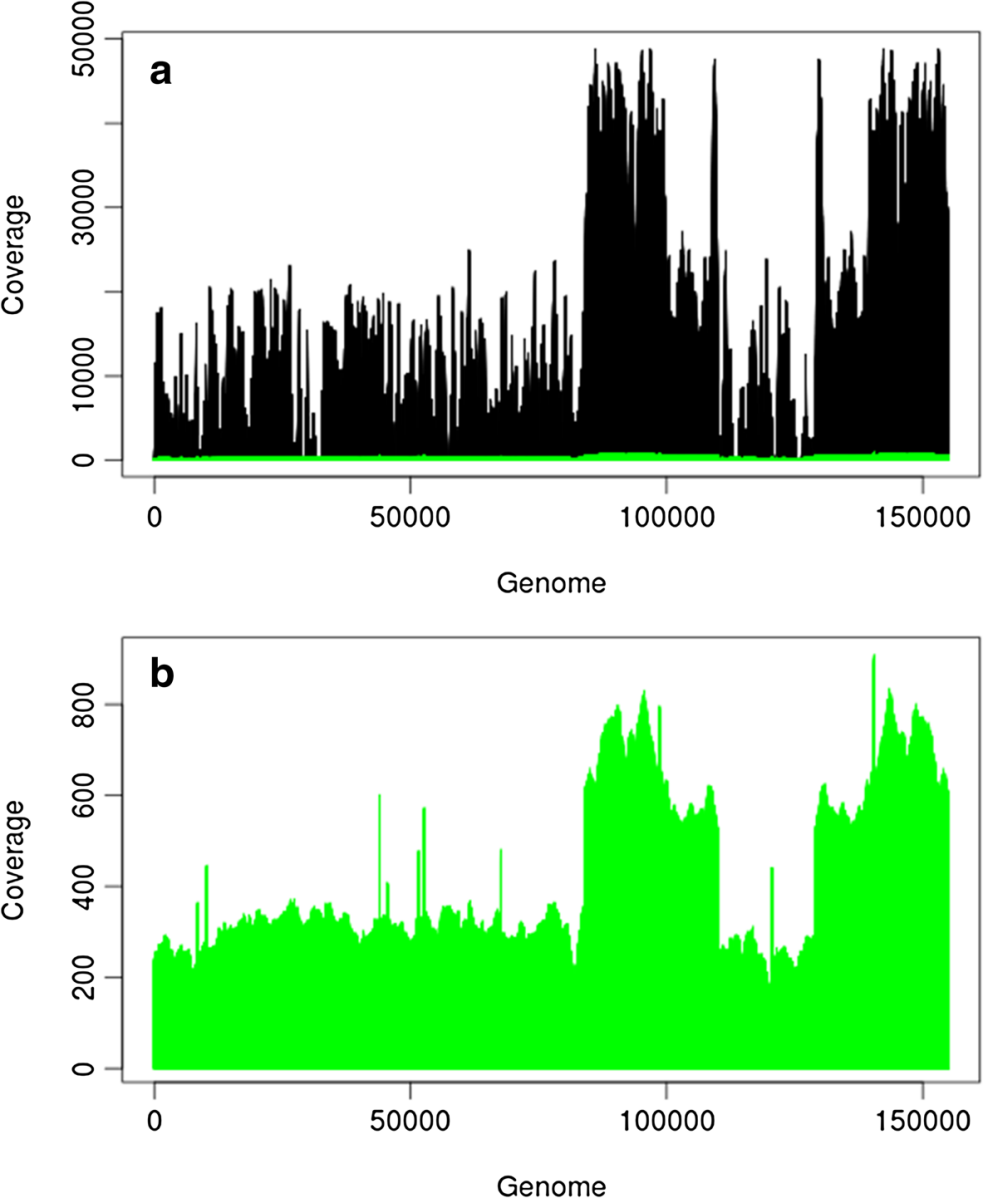
**Base-per-base coverage of the *P. micrantha *chloroplast genome.** Graph showing the base per base depth of sequencing coverage across the *P. micrantha* chloroplast genome with **(a)** Illumina (black) and PacBio (green) data and **(b)** PacBio data only, revealing a more uniform coverage of PacBio data across the genome despite the substantially lower depth of coverage, and regions of the genome with poor or zero coverage in the Illumina dataset. The two regions of significantly greater coverage in both datasets represent the two inverted repeat regions.

Both the PacBio and Illumina reads covered the majority of the *P. micrantha* chloroplast genome, with 100% and 99.6% of the genome covered by PacBio and Illumina respectively following alignment of all reads from each dataset to the assembled chloroplast consensus sequence using BLAT. The high percentage coverage of both datasets following the BLAT alignment supports the use of closely related chloroplast genomes to extract chloroplast-containing reads from the raw datasets generated from both the Illumina and PacBio platforms, and suggests this process did not bias the data towards longer PacBio reads. Low read coverage in certain regions of the Illumina assembly (Figure 2) meant that the seven contigs resolved covered just 90.59% of the chloroplast consensus sequence (Figure 1), whilst the PacBio data were significantly more evenly distributed (Figure 2) and were assembled into a single contig which formed the basis of the chloroplast consensus sequence presented here (Figure 1).

BLAT aligned a total of 25,384 reads containing a total of 49,654,764 bp from the PacBio RS dataset and 14,225,445 reads containing 1,411,774,265 bp from the Illumina dataset. Thus, the average depth of coverage of the *P. micrantha* chloroplast genome represented by the error-corrected PacBio RS data was 320×, whilst the average depth of coverage of the Illumina reads was 9,111×. Figure 2 shows the base per base coverage of the reads aligned by BLAT for both the PacBio and Illumina datasets across the *P. micrantha* chloroplast genome, showing a more uniform coverage of genome by the PacBio RS dataset.

**Figure 3:**
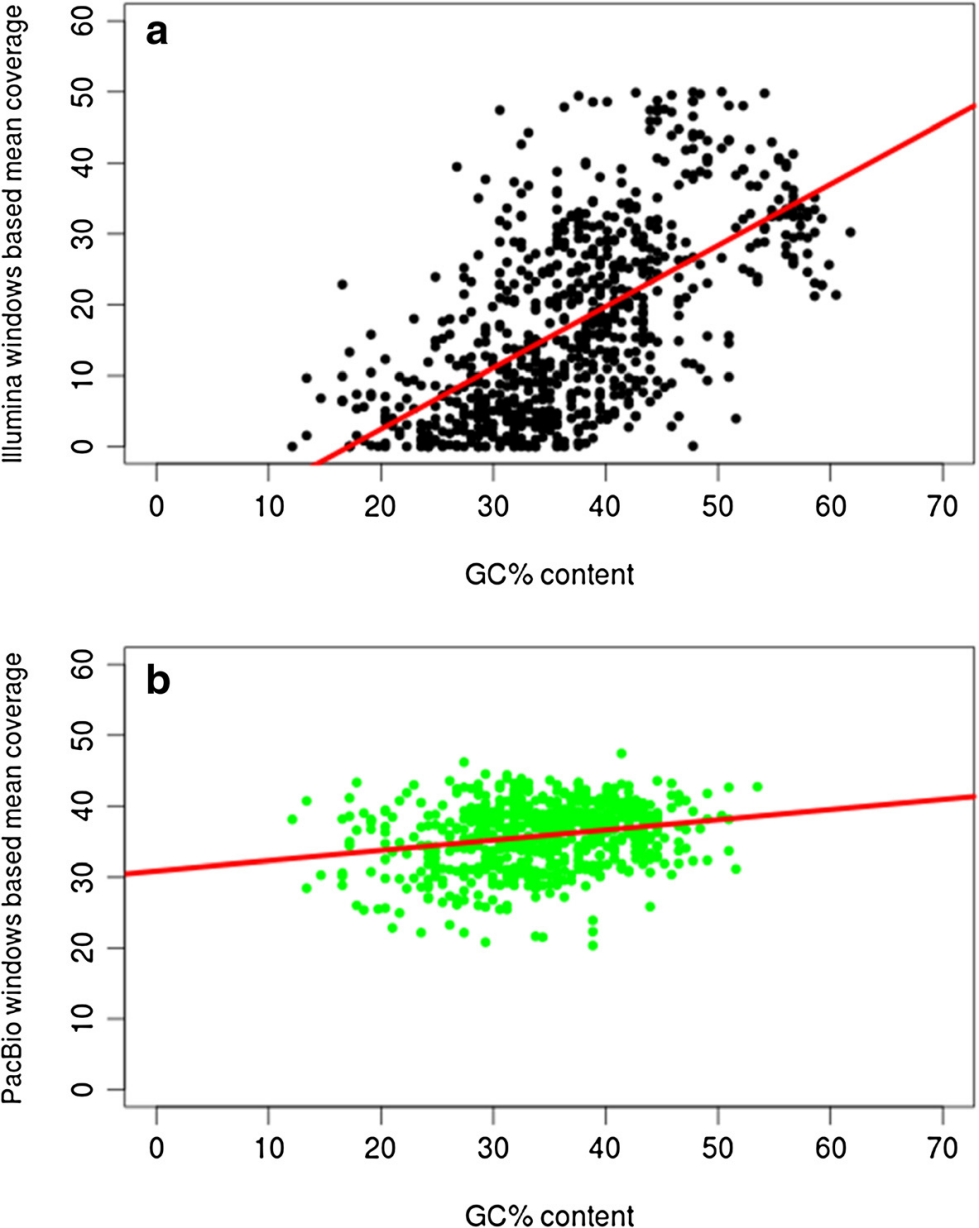
**Determination of percentage GC bias in the Illumina and PacBio datasets.** Percentage of mean depth of coverage across 987 windows of 157 nucleotides plotted as a function of percentage GC content for **(a)** Illumina (black) and **(b)** PacBio (green) data showing a much stronger positive dependency within the Illumina data (Pearsons correlation coefficient = 0.61 p-value = 2.2e-16) than in the PacBio data (Pearsons correlation coefficient = 0.23 p-value = 5.675e-09). For the purposes of the calculation, high coverage data from the two inverted repeat regions were excluded.

To determine whether a GC bias existed in the two sequencing datasets, the Pearson correlation coefficient was computed between mean coverage and percentage GC content in 987 contiguous non-overlapping windows of 157 nucleotides. For the purposes of the calculation, data from the two inverted repeat regions was excluded. The calculated Pearson correlation coefficients were 0.23 (p-value = 5.675e-09) and 0.61 (p-value = 2.2e-16) respectively for the PacBio and Illumina datasets. Thus, a much stronger positive dependency between the mean coverage against percentage GC content was observed in the Illumina dataset than in the PacBio data (Figure 3).

### Error rates

The mean pre-assembly error rate in the PacBio RS reads in comparison to the *P. micrantha* chloroplast consensus sequence was 1.3%, whilst the mean error rate in the Illumina reads was 0.117% compared to the chloroplast consensus sequence. Post-assembly, the two assemblies were generally in concordance however, 187 nucleotide sites could not be discriminated unambiguously in the Illumina assembly (two or more bases were called at each position). Performing error-correction prior to assembly using CORAL [14] on the Illumina reads did not help resolve the ambiguities at these 187 sites (data not shown). However, inspection of coverage and base calling at those sites in the PacBio RS data showed a clear single nucleotide consensus and thus all 187 nucleotides were resolved unambiguously in the chloroplast consensus sequence.

### Chloroplast genome assemblies at different depths of sequence coverage

To determine the effect of depth of sequence coverage on the assembly of the *P. micrantha* genome using PacBio RS data, a titration of sequence depths was performed with data sampled at 10×, 20×, 35×, 50×, 100×, 150× and 200× depth of coverage, following which the genome was assembled *de novo* from each dataset using the procedure described for the full datasets. Of the seven assemblies performed, five (from 200× to 35×) returned a single contig spanning the chloroplast genome, whilst the assembly performed at 20× returned four contigs spanning 95.6% of the genome and the assembly at 10× returned 14 contigs spanning 78.2% of the chloroplast genome. For comparison, Illumina data were sampled at the same seven depths of coverage as the PacBio data and assemblies were performed, however, none returned more complete assemblies than that performed with 9111× depth of coverage.

### Structural organisation of the *Potentilla micrantha* chloroplast genome

**Figure 4:**
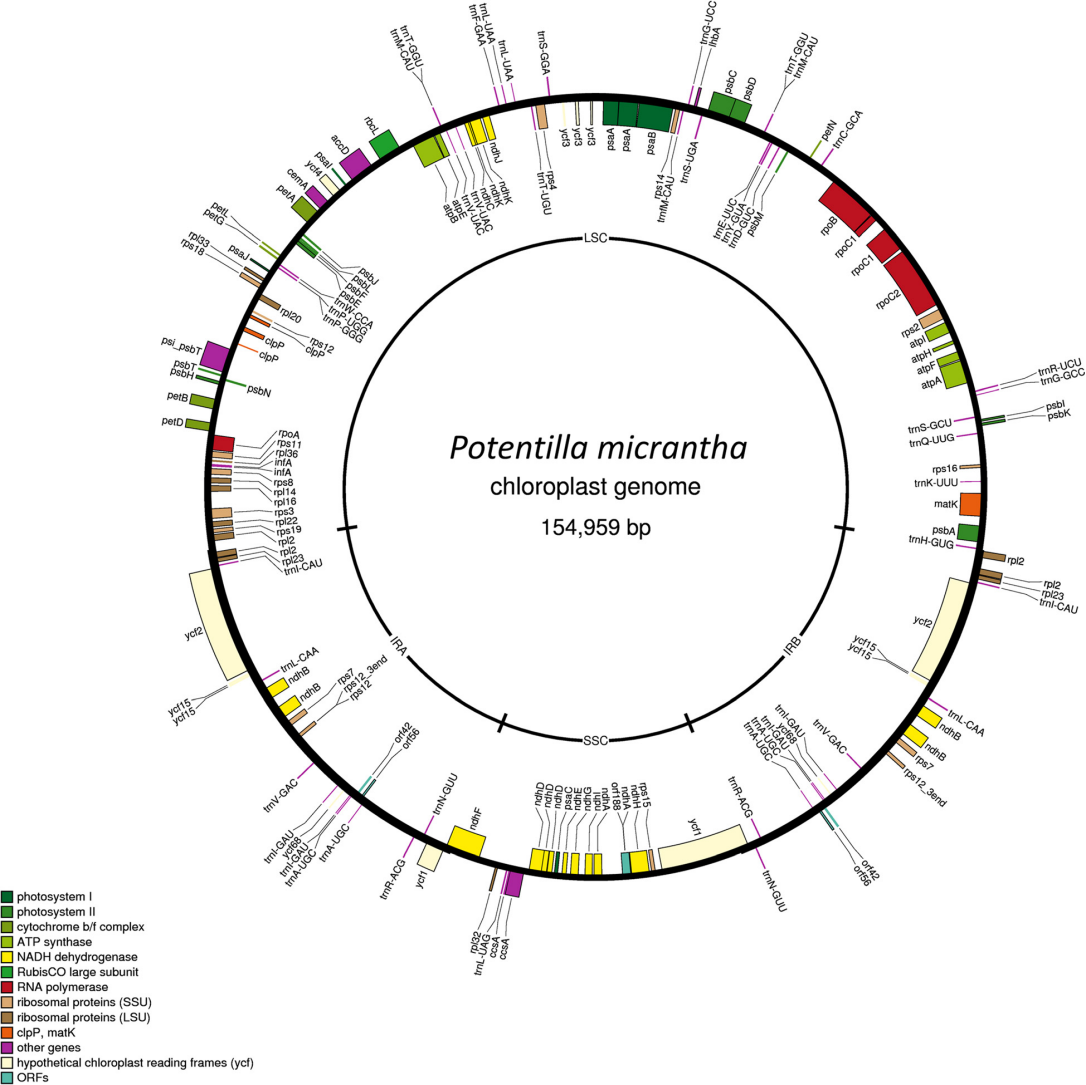
**The *P. micrantha *chloroplast genome sequence.** Structural organisation of gene content of the *P. micrantha* chloroplast genome detailing genes transcribed clockwise inside the circle and genes transcribed counter-clockwise outside the circle. Genes coloured according to functional categorisation, inner circle indicates mean percentage GC content across the genome**.** IRa and IRb denote inverted repeat regions, LSC and SSC denote long and short single copy regions respectively. Genome map plotted using OGDRAW [15].

The assembled chloroplast genome of *Potentilla micrantha* was 154,959 bp in length (Figure 4). The inverted repeats (IR) were 25,530 bp in length each, whilst the large single copy (LSC) and small single copy (SSC) regions were 85,137 bp and 18,762 bp in length respectively. The *P. micrantha* chloroplast contains 120 genes, 21 of which are duplicated in the IRs, giving a total of 141 genes of known function. Of these genes, 31 were tRNA coding genes, of which seven were located in the IR. A comparison with the *F. vesca* chloroplast genome sequence, the closest relative to *P. micrantha* for which a fully-sequenced chloroplast genome is available, revealed that the gene number and order within the genomes was identical between the two species.

Data relating to this project have been submitted to the ENA Sequence Read Archive of the EMBL database under the project accession number PRJEB4540.

## Discussion

Here we present the first report of the sequencing and *de novo* assembly of a chloroplast genome using the PacBio RS sequencing platform in which we recovered a single contig containing 154,959 bp that covered the entire *P. micrantha* chloroplast genome. To enable an evaluation of the relative performance of the PacBio RS sequencing platform for sequencing and *de novo* assembly of the *P. micrantha* chloroplast genome, we compared the results obtained to an assembly performed with a single library from the Illumina HiSeq2000 platform. Since the data from the two platforms were assembled by necessity using different assembly programs and assembly parameters, the results obtained clearly cannot be compared on a like-for-like basis, and the experimental design did not provide for the ‘optimal’ results that could be obtained for the assembly of a chloroplast genome using the Illumina HiSeq2000 platform. Nevertheless, Illumina data are recognised as being of immense utility to sequencing and *de novo* assembly of draft genome sequences, and thus, whilst the comparison is not intended to be a reflection of the performance of the HiSeq2000 platform *per se*, the resultant Illumina assembly provided a useful yardstick with which to judge the relative merits and short-comings of the PacBio RS sequencing platform.

Short-read sequencing platforms, including the Illumina HiSeq2000, derive sequencing reads from template DNA that has undergone pre-sequencing amplification by PCR [6]. This amplification step results in sequencing bias, and thus poor or no sequencing coverage in certain regions of the genome, and a strong positive correlation between %GC content and read coverage [16]. This lack of coverage is evident even when average depths of sequence coverage are high. Such bias leads to regions of no sequence coverage within sequencing datasets and thus assemblies that contain multiple small gaps, leading to a large number of contigs and scaffolds even in modest sized genomes such as those of bacteria [8,17] and chloroplast genomes [11]. In this investigation, the *P. micrantha* chloroplast genome was sequenced on the HiSeq2000 platform to an average depth of 9,111× from a single Illumina Truseq library, but despite this depth of coverage, there remained a total of 14,588 (9.41%) nucleotides of the genome which were not assembled from the Illumina data and thus seven contigs were recovered from the genome assembly. The gap regions contained a much lower average GC content than the entire chloroplast genome, in line with other studies that have reported a lower GC content in low coverage and gapped regions in Illumina assemblies [18] and reinforcing evidence of a strong positive dependency between coverage and GC content observed in the Illumina data set. In contrast, despite a lower depth of sequence coverage (320×) achieved following error-correction, data from the PacBio RS platform were assembled into a single contig spanning the entire *P. micrantha* chloroplast genome. Coverage of PacBio reads across the entire chloroplast consensus sequence was relatively even, demonstrating that data from this platform does not suffer from % GC and other context-specific biases affecting data produced by short-read ‘second-generation’ sequencing platforms [8]. Our data were also in accord with the recently reported findings of Tang et al [19] who recovered two contigs spanning the mitochondrial genome of tomato in an assembly using 122 × of PacBio data, in contrast to 835 scaffolds covering the same genome using 4098× of Illumina data, suggesting longer read length and less genome coverage bias can result in significantly longer contigs in *de novo* plastid genome assemblies.

It is possible that if multiple Illumina libraries, including mate-pair libraries and overlapping fragment libraries, were sequenced, then a single scaffold covering the chloroplast genome would have been recovered. However, due to the inherent biases in the PCR amplification performed prior to sequencing, it is likely that the scaffold would still have contained gaps associated with the regions of poor and no coverage as was found in this investigation and in other studies of chloroplast assembly using second generation sequencing platforms [11].

Indeed, assemblies performed following a titration of sequence depths for both PacBio and Illumina datasets demonstrated that the high depth of coverage of the Illumina dataset did not confound the assembly process, and no assembly at a lower depth of coverage performed better than the assembly utilising the entire Illumina dataset. PacBio assemblies at depths of coverage of 35× and above, recovered a single contig spanning the chloroplast genome, suggesting that *de novo* non-hybrid assemblies with PacBio data could be possible at relatively low depths of sequencing coverage.

Error-rates from single read data generated from the PacBio RS platform have been reported to be relatively high, in the region of 15.4 – 18.7% [5,6]. However, since sequencing errors are introduced randomly into the reads generated and are thus largely non-context specific [7], they are likely to have minimal effect on the final assembled sequence if sufficient depth of coverage is achieved and error-correction is performed prior to assembly. Since data generated from the Illumina HiSeq2000 platform has been established as the ‘gold standard’ for second-generation sequencing technologies, we evaluated the error-rate in the assembly of the PacBio RS data by comparison to Illumina data and where both assemblies resolved the same result for a nucleotide, we took this as an indication that the base had been called correctly in both assemblies. In this investigation, error rates of 1.3% were observed in the PacBio RS data following processing and error correction using HGAP [10] when compared to the chloroplast consensus sequence. Illumina sequencing data has been shown to contain non-random distribution of errors, with 3% of all error positions accounting for 24.7% of all substitution errors in one study [16] and no universal motif responsible for the occurrence of these error-prone positions. This type of error was observed at 187 nucleotide sites in the contigs derived from the Illumina assembly of the *P. micrantha* chloroplast genome in this investigation which despite high sequence coverage, returned ambiguous base calls following assembly. In all cases however, these ambiguous nucleotides were unambiguously called in the assembly derived from PacBio RS data as one of the alternative bases in the Illumina assembly. The PacBio and Illumina assemblies were concordant at all other bases within the assemblies, indicating that post-error correction and assembly PacBio data are potentially as robust as data derived from other sequencing platforms if sufficient depth of coverage is achieved to permit reliable error-correction. Indeed, recent reports suggest that with the latest chemistry and the most recent version of the HGAP algorithm, accuracy rates in PacBio RS datasets post-error-correction as high as 99.999% could be achieved [10]. It is important to highlight here however that the analyses performed for creating the consensus sequence favour the PacBio assembly since it contains more nucleotides than the Illumina assembly. Thus where no Illumina data were available for comparison, the PacBio data may contain a low percentage of errors that could not be verified in this study.

In previous studies, PacBio RS data have been reported to contain maximum read lengths of up to 23,000 nucleotides and median lengths of 2,446 nucleotides [5]. Such read lengths have been shown to significantly improve the quality of sequence assemblies when used for hybrid assemblies [8]. In this investigation, the maximum and mean un-corrected read lengths were 17,407 and 3,937 nucleotides respectively, with an average read length following error-correction of 1,902. The data generated using the PacBio RS platform covered a greater proportion of the chloroplast genome and was able to resolve the small percentage of ambiguities that were present in the Illumina assembly. Thus the data from the chloroplast assembly reported here supports previous findings that PacBio RS data can produce high quality sequence assemblies covering a greater proportion of the genome than can be achieved by Illumina sequencing alone [8].

PacBio RS data is significantly less expensive to generate than data from traditional Sanger sequencing, and reports indicate that for targeted exon sequencing, for use in genomic profiling of tumor biopsies, PacBio RS sequence data was in 100% concordance with traditional Sanger sequencing [20]. Additionally, other researchers demonstrated the utility of PacBio RS data for SNP validation in medical re-sequencing projects, where Sanger sequencing has traditionally been employed [7].

However, the additional read length of PacBio RS data comes at the cost of a higher cost per base than ‘second generation’ short read technologies [21], and higher single molecule error-rates necessitates the need for a greater depth of sequence coverage to be achieved to permit consensus accuracies of an acceptable level for *de novo* sequence assembly with currently available software. Additionally, since the PacBio sequencing platform performs real-time sequencing from single molecules, a greater quantity of DNA is required than second generation sequencing platforms, which could be a limiting factor for sequencing from organisms from which DNA is hard to obtain or which are difficult to culture. Despite the advantages to the use of PacBio RS sequencing data, and recent significant increases in throughput, the cost per base for *de novo* sequencing and assembly of larger genomes, such as those of plants are still significantly more expensive than data derived from the Illumina HiSeq platform [22]. Thus *de novo* assemblies of the genomes of minority species at the time of writing may be best served through the combination of PacBio data with data from other platforms. Koren et al. [8] demonstrated that the addition of a modest amount of Illumina error-corrected PacBio data to supplement 454 sequencing data from multiple libraries resulted in a 32% increase in N50 sizes in the parrot (*Melopsittacus undulatus*) genome sequence assembly and other researchers have demonstrated the utility of PacBio sequence data for gap filling and genome finishing [23]. The data presented here support the findings of those previous studies and illustrate the power and utility of PacBio RS sequencing data for sequencing and *de novo* assembly, as well as demonstrating that despite high initial single read error rates, following error-correction and assembly, the data produced by the platform are robust and reliable.

## Conclusions

As part of an on-going effort to sequence the nuclear genome of *P. micrantha*, we are employing PacBio sequence data in combination with Illumina small insert and mate pair sequencing libraries and initial data suggest that, as with the chloroplast data presented here and by other authors, PacBio RS sequencing data show great promise in scaffolding, gap filling and genome sequence finishing. In addition, if the current trend in increased throughput and reliability continue, it is reasonable to speculate that the technology may be able to deliver affordable high quality finished genomes for a variety of eukaryotic organisms.

## Methods

### Plant material

A single accession of *Potentilla micrantha* was collected at Avala, Beli Potok, Serbia. It was dug from the soil in August 2012, retaining as much of the root system of the plant as possible, repotted into standard potting compost and maintained at the Vigalzano experimental station of the Edmund Mach Foundation (FEM), Pergine, Italy, where it was grown under supplementary lighting maintaining a 16 h photoperiod and a constant 20°C to promote vegetative growth.

### DNA extraction

Unexpanded young leaves of the *P. micrantha* accession were removed from the plant and freeze-dried for 48 hours. Leaf tissue was then ground using a Retsch mixer mill (Retsch) in a 2 ml microcentrifuge tube with a tungsten carbide bead for 60 sec until finely powdered. DNA was extracted using a ‘user-adapted protocol’ with Qiagen genomic tips (Qiagen) with minor modifications. Briefly, powdered leaf tissue was re-suspended in 15 ml of a lysis buffer containing 20 mM EDTA, 10 mM Tris Cl (pH7.9), 0.5 mg/ml driselase (Sigma), 1% Triton X-100, 500 mM Guanidine-HCl, 200 mM NaCl in a 50 ml Falcon tube and the suspension was incubated at 45°C for 2 h in a heated mixing block at 450 rpm. Next, 300 μg of RNase A (Qiagen) was added and the sample incubated at 37°C for a further 30 minutes. A total of 12 mg of proteinase K was added and the sample incubated for a further 2 h at 450 rpm at 50°C. Following incubation, the sample was transferred to eight 2ml microcentrifuge tubs and centrifuged at 15,000 × *g* for 30 minutes. Equal measures of the eluate were then transferred to four 100/G Genomic tips (Qiagen) equilibrated with 3 ml of buffer QBT (Qiagen) and allowed to pass through the column by gravity flow. Each tip was washed twice with 10 ml of buffer QC (Qiagen) following which, DNA from each column was eluted with 5 ml buffer QF (Qiagen). DNA was precipitated in a 1.5 ml microcentrifuge tube by adding 0.7 volumes room-temperature isopropanol and centrifugation at 15,000 × *g* until all DNA was precipitated in a single tube. DNA was washed three times with 70% ethanol kept at −20°C, air-dried and resuspended in 200 μl tissue-culture grade water (Sigma). DNA quality, quantity and integrity were determined through spectrophotometry using the Nanodrop 8000 platform (Thermo Scientific), fluorospectrometry using the NanoDrop 3300 fluorospectrometer platform (Thermo Scientific), and agarose gel electrophoresis. High-molecular weight DNA with an OD 260/280 above 1.9 and OD 260/230 above 1.9 and a yield of at least 10 μg was sent for sequencing.

### Library construction and sequencing

#### Pacific Biosciences PacBio RS

A total of 10 μg of DNA was sent lyophilized to the GATC Biotech genomics sequencing facility at Lake Constance, Germany, where a single 10 kb SMRT-bell sequencing library (Pacific Biosciences) was constructed. DNA was used to construct a 10 kb SMRT-bell library by GATC Biotech following the protocol described in Quail et al. [21]. The SMRT-bell library was sequenced using 26 SMRT cells (Pacific Biosciences) using C2 chemistry and 2 × 45 minute movies were captured for each SMRT cell using the PacBio RS (Pacific Biosciences) sequencing platform. Primary filtering was performed on the RS Blade Center server following which secondary analysis was performed using the SMRT analysis pipeline version 1.4.

#### Illumina HiSeq2000

A total of 5 μg of DNA from the same extraction was sent lyophilized to TGAC, Norwich, UK for sequencing using the Illumina Hiseq2000 sequencing platform. A single Truseq library was constructed from the DNA containing a 450 bp insert size following standard Illumina protocols. A PhiX kit v2 library (Illumina) was spiked into the sample library at a proportion of 1%, and the library was sequenced without indexing on a single lane of a HiSeq2000 flow-cell for 2 × 101 cycles.

### Extraction of chloroplast reads from Illumina sequence data

SMALT (http://www.sanger.ac.uk/resources/software/smalt/) was used with default parameters to filter the PhiX internal control from the total Illumina data using the PhiX genome sequence, along with other contaminating sequence using the NCBI UniVec database. SMALT was then used to extract chloroplast DNA reads using the *Fragaria vesca* (EMBL accession JF345175), *Malus × domestica* (http://www.rosaceae.org), *Nicotiana tabacum* (EMBL accession Z00044), *Glycine max* (EMBL accession DQ317523), *Medicago truncatula* (EMBL accession AC093544), *Prunus persica* (EMBL accession HQ336405), *Populus alba* (EMBL accession AP008956) and *Solanum lycopersicum* (EMBL accession AM087200) chloroplast genomes as queries. Only reads with percentage of similarity over 90% were extracted from the dataset and considered as chloroplast material. Quality trimming of the Illumina data was performed with the windowed adaptive trimming tool Sickle (https://github.com/najoshi/sickle), using q = 30 as the threshold for trimming based on average quality in the sliding window and l = 50 as the threshold to keep a read based on length after trimming.

### Illumina data assembly

Illumina data were assembled with AbySS [24] using default parameters. Assemblies were performed using all odd *k*-mer lengths between 17 and 91. Assembly N50 sizes and total numbers of contigs were evaluated and 20 assemblies giving the most consistent results (*k*-mer lengths of 19, 21, 25, 27, 31, 33, 39, 41, 45, 47, 51, 53, 59, 61, 65, 67, 71, 73, 77, 81) were retained. Subsequently, the resulting assemblies were clustered using CD-Hit [25] using a threshold of 100% to remove redundant contigs, and the unique contigs were merged using the minimus2 application of the AMOS 3.1.0 assembly package [26] running default parameters. The duplication of the IR was resolved manually through identification of the IR boundaries in the *Potentilla* assembly and comparison to the IR region of the closely-related *Fragaria* chloroplast genome sequence as has been performed in other species [13], to produce an assembly containing two complete IRs.

### PacBio RS read error-correction, chloroplast read extraction and data assembly

Pre-assembly error correction was performed with the hierarchical genome assembly process (HGAP) of SMRT Analysis version 1.4 (Pacific Biosciences, USA) using default parameters. Full details of the HGAP workflow are detailed in Chin et al. [10]. From the error-corrected data, reads containing chloroplast genomic sequence were extracted using BLAT, as SMALT does not handle long read data, following the procedure described above for the Illumina dataset except that due to a potentially higher error rate in the PacBio data, all matches with other chloroplast genomes were retained. Error-corrected chloroplast reads were then assembled using Celera Assembler [8,27]. Following assembly, chloroplast contigs were merged using the minimus2 application of the AMOS 3.1.0 assembly package [26] running default parameters and for the titration of depths of sequence coverage, SeqMan (LazerGene) using a match size of 5, a minimum match percentage of 95 and a minimum sequence length of 1000. The identical section of the IRs was resolved manually, to produce a contig containing two complete IRs in line with other published chloroplast genomes, spanning the entire length of the *P. micrantha* chloroplast genome.

### Formation of the *P. micrantha* chloroplast consensus sequence

The *P. micrantha* chloroplast consensus sequence was formed from the single PacBio RS sequence contig from which the two IR repeats had been resolved as described above. Illumina contigs were aligned against the PacBio consensus sequence using BLAST and 187 incongruities in the Illumina data were identified. Following BLAST, both Illumina and PacBio reads were aligned to the chloroplast reference sequence using SMALT and BLAT respectively and the incongruities were resolved by taking the majority-rules nucleotide from the two alignments at these sites. In this way, all incongruities in the Illumina contigs were resolved.

### Genome coverage and GC bias

To evaluate the completeness of coverage across the *P. micrantha* chloroplast genome of the Illumina and PacBio datasets, the depth and breadth of genomic coverage obtained with each platform were analysed by plotting coverage plots as described in [28] from data aligned to the chloroplast consensus sequence of the chloroplast genome using BLAT using default parameters [29]. The nucleotides in the complete chloroplast genome were divided into 987 windows of 157 nucleotides each. For each window the percentage GC content and the mean coverage of the Illumina and PacBio datasets was plotted using RStudio 2.13.1 and a Pearson correlation coefficient was computed for both datasets against percentage GC content using custom scripts (Additional file 1).

### Calculation of errors in PacBio and Illumina datasets

To calculate the relative error rates in the PacBio data from the BLAT alignment of the PacBio data against the chloroplast consensus sequence, the number of mismatches in the alignment was summed and divided by the total number of nucleotides in the alignment using a custom Python script (Additional file 2). To calculate the relative error rates in the raw Illumina data, a SamTools pile-up was created using SAMtools-0.1.19 from the SMALT alignments of the raw Illumina data against the chloroplast consensus sequence. The number of mismatches and the mean error rate for each read compared to the chloroplast consensus sequence was then calculated based on the total number of aligned nucleotides in the SamTools pile-up using a custom Python script (Additional file 3).

### Gene annotation and comparison with the *Fragaria vesca* chloroplast genome

Gene prediction was performed on the FASTA file containing the complete *P. micrantha* chloroplast genome sequence using DOGMA [30] with default settings. Comparison of gene number and order between the *P. micrantha* and *F. vesca* chloroplast genomes was performed manually using *F. vesca* gene predictions performed by DOGMA.

All command line references for data processing and assembly are given in Additional file 4.

## Competing interests

The authors declare that they have no competing financial interests.

## Authors’ contributions

MF carried out the data analysis and co-authored the manuscript. MM carried out data analysis and co-authored the manuscript. JAW conceived the experiments, carried out data analysis and co-authored the manuscript. NS collected plant material, conceived the experiments and critically evaluated the manuscript. VS collected plant material and provided valuable advice and discussion. LG managed the plant collections and carried out experimentation. RVi critically evaluated the manuscript and contributed valuable discussion. DC advised on data analysis and critically evaluated the manuscript. RVe advised on data analysis and critically evaluated the manuscript. AC advised on data analysis and critically evaluated the manuscript. DJS conceived the experiments, carried out experiments and data analysis and authored the manuscript. All authors read and approved the final manuscript.

## Supplementary Material

Additional file 1 R scripts used to plot windows-based mean coverage against GC content in the PacBio and Illumiona datasets. Click here for file

Additional file 2 Python script used to calculate the mean error rate in the error-corrected PacBio reads from the BLAT alignment of the data against the chloroplast consensus sequence. Click here for file

Additional file 3 Python script used to calculate the mean error rate in the Illumina raw reads from the SMALT alignment of the data against the chloroplast consensus sequence. Click here for file

Additional file 4 Command line references for data processing and assembly performed in this study. Click here for file
