# Supplementary material for: An evaluation of the PacBio RS platform for sequencing and de novo assembly of a chloroplast genome
Source: BMC Genomics. 2013 Oct 1;14:670. doi: 10.1186/1471-2164-14-670 (PMC3853357; doi:10.1186/1471-2164-14-670)
Supplement: Additional file 4 — Command line references for data processing and assembly performed in this study. [file 1471-2164-14-670-S4.docx]

**ABySS 1.3.4**

ABySS-PE: all default parameters were used. *k*-mer size was changed to each *k*-mer size stated in the methods section.

**SMALT version 0.7**

Smalt index: All defaults were used. *k*-mer size and step size were as follows: -k 5 –s 2

Smalt map: All default parameters used

**SICKLE 1.2**

Sickle pe: Quality threshold and read length parameters were: -q 30 –l 50

**CD-Hit 4.5.4**

cd-hit: 50% identity threshold used: -c 0.5

**BLAT 35x1**

All default parameters used

**Celera Assembler 7.0**

runCA: All default parameters were used

**AMOS 3.1.0**

Minimus: all default parameters were used

**Lazergene 7.0**

SeqMan: match size 5 minimum match percentage 95 minimum sequence length 1000

**SAMtools-0.1.19**

samtools view: -bSh

samtools sort: all default parameters were used

samtools mpileup: all default parameters were used
